# Impact of Health Literacy in Patients with Chronic Musculoskeletal Disease–Systematic Review

**DOI:** 10.1371/journal.pone.0040210

**Published:** 2012-07-06

**Authors:** Yoon K. Loke, Ina Hinz, Xia Wang, Gill Rowlands, David Scott, Charlotte Salter

**Affiliations:** 1 Norwich Medical School, University of East Anglia, Norwich, Norfolk, United Kingdom; 2 Institute of Primary Care and Public Health, London South Bank University, London, United Kingdom; Marienhospital Herne - University of Bochum, Germany

## Abstract

**Objectives:**

To estimate the prevalence of low health literacy, and evaluate the impact of low health literacy on outcomes in patients with chronic musculoskeletal conditions.

**Data Sources:**

We searched Embase, Pubmed, PsycInfo, and CINAHL in January 2011 for relevant studies, restricted to English-language articles.

**Study Selection and Data Extraction:**

Studies were included if they measured health literacy and/or reported on the link between outcomes and health literacy levels in patients with osteoporosis, osteoarthritis, or rheumatoid arthritis. We assessed risk of bias from participant selection, methods of measuring health literacy and functional outcomes, missing data, and potential for confounding.

**Data Synthesis:**

We reviewed 1863 citations and judged 8 studies to be relevant. Most were cross-sectional in nature, and five were based in the United States. Diversity in measurements, participant characteristics, and settings meant that results had to be synthesized narratively. Prevalence of low health literacy varied from 7% to 42%. Of the five studies that reported on musculoskeletal outcomes, only one showed an association (unadjusted) between low health literacy and greater pain and limitations in physical functioning. However, other studies, including those with multivariate analyses, found no significant relationship between health literacy and measures of pain or disease specific questionnaires. One clinical trial found short-term improvements in the mental health of patients with musculoskeletal conditions after an intervention to improve health literacy.

**Limitations:**

Most of the studies were cross-sectional in nature, which precludes interpretation of a causal relationship. The sample sizes may not have been sufficiently large to enable detection of significant associations.

**Conclusions:**

The current evidence does not show a consistent association between low health literacy and poorer functional outcomes in patients with chronic musculoskeletal conditions. In the absence of a definite link, efforts to develop interventions to improve health literacy would not necessarily improve health service or patient-related outcomes.

## Introduction

The Institute of Medicine Committee for Health Literacy considers health literacy to be “the degree to which individuals have the capacity to obtain, process, and understand basic information and services needed to make appropriate decisions regarding their health”. [1] Older patients with low health literacy and cancer show less knowledge of their condition and its management, and have more difficulty making informed choices. [2] Low levels of health literacy are linked with increased hospital admissions and mortality, [3] whilst higher health literacy has been shown to be associated with greater health knowledge and self-confidence. [4].

Health literacy is considered to encompass three different levels. Functional health literacy refers to the basic reading and writing skills to manage health information in common daily settings, whereas the interactive and critical levels involve more advanced cognitive skills (e.g. improved ability to appraise new information and act independently, perhaps to influence change at personal and community levels). [5] Validated tools such as the Rapid Estimate of Adult Literacy in Medicine (REALM) or the Test of Functional Health Literacy in Adults (TOFHLA) can be used to measure health literacy. Components of the REALM include word recognition and pronunciation, whereas the TOFHLA evaluates reading comprehension and numeracy. [6] Both these tools are primarily aimed at functional health literacy. [7].

The complexity and potential toxicity of treatments, effects on quality of life and activities of daily living mean that low health literacy may have an important impact on patients’ disease severity and adherence to treatment. Low levels of health literacy may potentially cause patients with chronic musculoskeletal conditions to face difficulties in use of health services, and to possibly experience poorer control of their illness. For instance, a study from a specialist rheumatology clinic in Glasgow, United Kingdom found that patients with low health literacy levels had significantly more clinic visits than those with higher health literacy. [8].

The World Health Organization considers chronic musculoskeletal conditions to be a major source of morbidity and disability, with substantial adverse economic impact on healthcare resources, and the patient’s ability to earn a living. [9] Here rheumatoid arthritis, osteoarthritis, and osteoporosis are listed as the chronic rheumatic conditions with the greatest impact on society. If low health literacy was found to have an important association with greater health service use and poorer disease states, then efforts could be focused on tackling the underlying causes, and providing interventions to help improve health literacy. Two systematic reviews have reported some association between health literacy and general health outcomes, [10], [11] but there has not yet been any comprehensive review of health literacy and disease outcomes in patients with musculoskeletal illnesses. Hence, the aim of this present work was to review systematically the prevalence of low health literacy, and the associated healthcare outcomes in older patients affected by these chronic musculoskeletal conditions.

## Results

**Figure 1:**
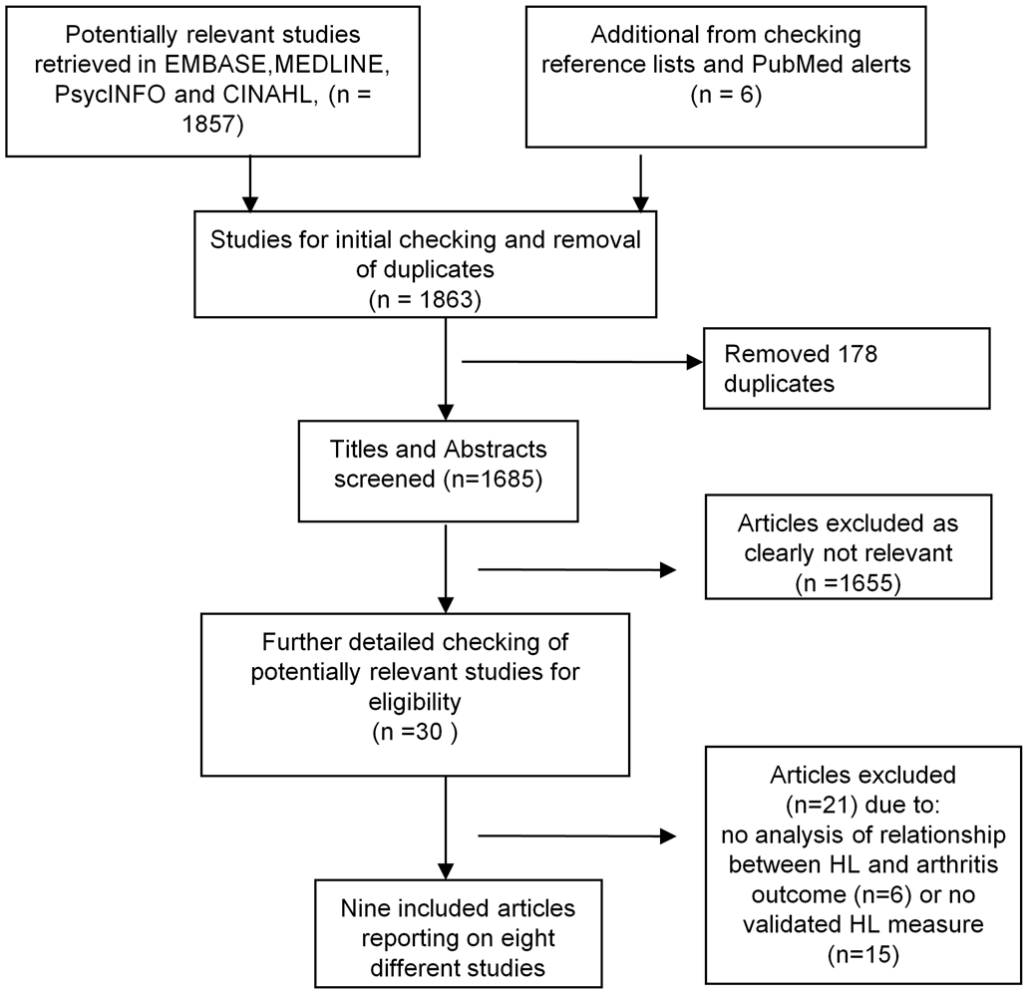
Flow chart of study selection.

The initial search yielded 1863 citations, and various studies were excluded through the screening and selection process (Figure 1).

### Characteristics of Studies

We included 9 articles reporting on eight different research studies. [12], [13], [14], [15], [16], [17], [18], [19], [20] Due to significant heterogeneity of study design, participants, and outcome measures, the individual studies were compared in a narrative manner (Tables 1 and 2).

**Table 1:** Study design and characteristics.

Study	Design; country and setting	Sample size	Mean age (yrs)	Participants
Bhat et al.(2008) [12]	Cross-sectional and longitudinal analysis ofcommunity-based RCT participants, NorthCarolina, USA.	447	69	Sedentary adults with arthritis, taking part in exercise orcognitive behavioural intervention trials.
Buchbinderet al. (2006) [13]	Cross-sectional study in communityrheumatology practice, Melbourne, Australia.	80	60	Consecutive patients aged >18 y with stable,well-controlled RA, attending regular review.
Hirsh et al.(2010) [15],Hirsh et al.(2011) [16]	Cross-sectional study in urban rheumatologyclinic in USA	110	53	English-speaking adult patients with RA, and no evidenceof uncontrolled psychiatric illness or visual impairment.To reduce selection bias, investigators did not use writtenmaterial during recruitment.
Gordon et al.(2002) [14]	Cross-sectional study in tertiary referral centrein Glasgow.	123	56 (median)	Adults with 4 consecutive attendances at clinic forrheumatoid arthritis
Kim et al. (2009) [17]	Cross-sectional study of community centresin Korea	103	72	Age >60 years with no apparent cognitive or visualimpairment, attending senior welfare centres.
Rudd et al.(2009) [19]	RCT of an educational intervention to reduceliteracy barriers of patients with inflammatoryarthritis in an urban teaching hospital, USA	127 (63 in standard care,64 individualizedor Plain English arm)	58.5+/−13.8 years	Adults >18 y, with rheumatoid arthritis/psoriatic arthritis/inflammatory polyarthritis withat least one visit with rheumatologist, not medical professionals, withoutpost-graduate degree, no visual impairments, comfortable withspoken/written English; ratio of 3∶1 patient with low: higheducation attainment.
Quinlan et al.(2010) [18]	Cross-sectional study of outpatients fromacute care facility in New York city	125	58	Outpatients at musculoskeletal clinic, 80% private patients,20% public patients.
Swearingenet al. (2010) [20]	Cross-sectional study at academicrheumatology clinic, Nashville USA.	194	57	Outpatients with various rheumatic diseases

**Table 2:** Study outcome and results.

Study	Prevalence of Low Health Literacy	Health Literacy Measure	Other Outcome Measures	Results
Bhat et al. (2008)[12]	REALM –89/447 (20%)	REALM	HAQ, and VAS for pain,fatigue, and stiffness.	Multivariate analysis found that levels of health literacy (low or adequate) did not have significant relationship with baseline or post-intervention health status i.e. disability (HAQ), pain, fatigue or stiffness.
Buchbinder et al. (2006) [13]	7/80 (9%) on TOFHLA and 8/80on REALM (10%).*	TOFHLA, REALM	Educational level	Significant numbers of patients with RA have low health literacy.
Hirsh et al. (2010) [15], Hirsh et al. (2011) [16]	35% inadequate or marginal(S-TOFHLA), 49% below highschool reading level (REALM),30% ‘somewhat confident’ orless (SILS).	2010: S-TOFHLA, REALM, SILS	Global assessment of disease state (based on MD-HAQ and DAS-28 scores), completed by patients and by health professionals.	Low health literacy independently associated with the extent of discrepancy between patient’s own assessment of health status as compared to physician’s assessment (p<0.001), with lower S-TOFHLA scores associated with wider gap between assessments (after adjustment for covariates). Limited health literacy REALM and TOFHLA) not associated with disease activity (DAS-28), including stiffness, pain, or steroid/biologic use.
Gordon et al. (2002) [14]	18/123 (15%) had REALMscores ≤60.	REALM	Interview and case record review for demographic data and clinic attendance. Functional status throughHAQ and HAD.	Low health literacy associated with anxiety (p = 0.011) and socioeconomic deprivation (p = 0.0064). Those with low literacy had more outpatient clinic visits than age and sex-matched literate (6 vs 2 visits/year) but HAQ score, sex, age, disease duration, joint replacement and number of previous disease modifying drugs not significantly different,
Kim et al. (2009)[17]	43 subjects (42%) had ascore<5, indicating lowhealth literacy	Korean version of TOFHLA	Questionnaire on educational level, and comorbidconditions	Individuals with low health literacy had significantly higher prevalence of arthritis than literate group (51% vs. 22%, p = 0.003). After adjusting for age and income, those with low health literacy had lower levels of physical function and subjective health, and more pain and limitations in activity.
Rudd et al.(2009) [19]	21% in the standard care and16% in intervention group werebelow high school levelreading	A-REALM (arthritis specific)	Adherence-scale, Lorig’s self-efficacy scale, Medical intervention satisfaction scale, appointment keeping, HAQ, mental health (subsection of SF-36)	Mental health significantly better (p = 0.04) in the intervention group at 6 months post intervention (but not significant at 12 months, p = 0.11); self-efficacy significantly better at 12 months (p = 0.04); other primary outcomes (adherence, satisfaction, appointment keeping) did not show any difference.
Quinlan et al. (2010) [18]	4/125 inadequate,5/125 marginal	Modified brief version of TOFHLA	Morisky Medication Adherence Scale, and adapted Arthritis Knowledge Questionnaire	In multivariate regression, level of health literacy was predictive of health knowledge (p = .002). However, level of health literacy was not a predictor of adherence (p = .896) after controlling for health knowledge and patient covariates.
Swearingen et al. (2010) [20]	35/194 (18%) with REALM;24% with A-REALM	REALM, A-REALM (arthritis specific)	HAQ and MD-HAQ; physical function, pain, global status and laboratory tests	Univariate analysis: those with low health literacy had significantly poorer global status (p<0.05) and non-significantly poorer physical function, pain, fatigue, and inflammatory markers

**Abbreviations:** DAS 28 = 28 joint count disease activity score- measures swelling and tenderness in 28 joints; HAD = Hospital Anxiety and Depression questionnaire; HAQ = Health Assessment Questionnaire; health literacy = health literacy; MD-HAQ = multidimensional health assessment questionnaire; VAS = Visual Analogue Scale where an X on a line indicates a score between ‘very well’ and ‘very poorly’; RA = Rheumatoid Arthritis; SILS = single item literacy screener “how confident are you filling out medical forms by yourself?”.

Five of the eight studies were conducted in the USA, [12], [15], [18], [19], [20] and there was one study each from Australia, South Korea, and the UK. [13], [14], [17] Studies’ sample size ranged from 80 to 447 and participants’ mean age ranged from 55.8 to 78 years. Three of the studies focused on rheumatoid arthritis, [13], [14], [15] one enrolled patients with a range of arthritic conditions (including rheumatoid, psoriatic and other inflammatory), [19] while the other studies looked at patients attending Rheumatology/musculoskeletal clinics whose diagnoses were less clearly defined.

Measurements of health literacy varied, with some studies using more than one health literacy tool. Five studies used the REALM, four used the TOFHLA or its abbreviated version, two used an arthritis-specific version of REALM, and one used the Single Item Literacy Screener (SILS).

### Prevalence of Low Health Literacy

There was considerable variation in the prevalence of low health literacy amongst the eight studies, with a range from 7% to 42%. The lowest prevalence of 7% was seen in a group of patients attending an acute care facility in New York, [18] whereas the highest prevalence of 42% was recorded in older adults attending senior welfare centers in South Korea. [17].

### Relationship between Low Health Literacy and Disease Specific Measures

Five studies looked for a potential association between low health literacy and clinical measures that were specific for rheumatic conditions, but only one study appeared to suggest a clear relationship. [17] Kim’s study found that those with lower health literacy had poorer physical function, with more pain and limitation. [17].

In contrast, multivariate analysis in two studies did not show any significant relationship between level of health literacy and rheumatoid arthritis Disease Activity Score (DAS-28), [15] or specific symptoms of pain, fatigue or stiffness. [12], [16] Equally, univariate analysis from two different rheumatology clinics did not show a significant association between health literacy and outcome measures such as pain, fatigue, inflammatory markers, joint replacements, or number of rheumatic drugs used. [14], [20].

### Relationship between Health Literacy and Other Dimensions of Health

There was considerable heterogeneity in the findings from six studies reporting on overall physical wellbeing, or other dimensions such as mental health in patients with chronic musculoskeletal conditions. Multivariate analysis in one study found no significant relationship between health literacy and health status based on the Health Assessment Questionnaire (HAQ), [12] while multivariate analysis in Hirsh’s study suggested a non-significant association between REALM or S-TOFHLA with poorer general health on multi-dimensional HAQ (MD-HAQ). [16] However, this study demonstrated a significant association between overall health and SILS (a single item screening tool based on the question “How confident are you filling out medical forms by yourself?”). [16].

Hirsh also reported considerable discrepancy between the patients’ own assessments of health status as compared to the physicians’ assessments. [15] This gap between patient and physician judgment (where patients considered themselves to be in a poorer health state than what their physicians believed) was most prominent in those with low S-TOFHLA scores. In two other studies with potential for confounding, low health literacy seemed to be significantly associated with poorer global status or subjective health. [17], [20].

Mental health in patients with rheumatoid arthritis conditions was reported in two studies. [14], [19] In a cross-sectional study, Gordon et al. found that low health literacy was associated with greater levels of anxiety. [14] A randomized controlled trial of an educational intervention to reduce literacy barriers demonstrated significant improvement in mental health at 6 months, but not at 12 months, when compared to control patients. [19] This trial found that the health literacy intervention did not significantly improve medication adherence, appointment keeping, or overall health status (HAQ) as compared to controls.

### Relationship between Health Literacy and Health Service Outcomes

We identified two articles reporting on the association between low health literacy and outcomes related to delivery or use of health care. [14], [18] In Gordon’s study, low health literacy was associated with more frequent hospital attendance (6 visits per year as compared to two per year in age and sex-matched patients with higher health literacy). [14] Patients with low health literacy were also from lower socio-economic groups, and had greater levels of anxiety. In another study, Quinlan used multivariate analysis to show that health literacy had a significant link to health knowledge, but that health literacy (as an independent variable) did not predict self-reported medication adherence in patients attending a musculoskeletal clinic. [18].

### Risk of Bias (Table 3)

Seven of the eight studies were cross-sectional in design, [12], [13], [14], [15], [16], [17], [18], [20] with the exception being a randomized controlled trial evaluating an educational intervention for low literacy. [19] Selection bias was a potential problem in certain studies that required patients to be regular attendees with stable disease, or to have at least 4 consecutive attendances. [8], [13] The number of patients declining to participate, and their socio-demographic characteristics was seldom described, with one study stating that around 30% of eligible participants had declined to take part. [15].

**Table 3:** Risk of Bias.

Study	Loss to follow-up	Statistical adjustment	Limitations
Bhat et al.(2008) [12]	17% (n = 121) did not do the REALM-not reported why, these might possibly be themost illiterate people	Age, gender, race, BMI, educational, marital and work status.	Data from two RCTS was combined but one was of 8, the other of 20 weeks duration, one was a behavior modification the other an exercise intervention; 6-months follow up data was only collected in the intervention group not in the control group.
Buchbinderet al. (2006)[13]	3 of 83 refused to participate, (not able toread well = 2, reason not given = 1). Of the 80 participants,1 did not attempt the TOFHLA and 15 did not do the TORCH-reasons not reported.	Not reported	Limited generalisability as sample was regular attendees at private clinic. Two patients unable to participate due to inability to read, while one patient did not complete TOFHLA.
Hirsh et al.(2010) [15], Hirshet al. (2011)[16]	118 recruited but 8 withdrew (due to illiteracy = 2, reasons not given = 6). Of the remaining 110, 2 did not complete patient assessment.	Multivariate analysis adjustedfor use of biologic agents,education, sex, age	About 70% of eligible patients agreed to participate, but 8/118 then withdrew, 2 due to illiteracy, and 1 due to poor command of English. 2/110 did not complete patient assessment.
Gordon et al. (2002) [14]	4 of 127 refused to take part (3 of thosesaid they were unable to read).	Not reported	Limited generalizability as sample was from tertiary care centre. Four subjects refused to participate (three of whom could not read)
Kim et al.(2009)[17]	7 of 110 subjects agreeing to participate(survey response rate 65%) in the studywere excluded because of vision problemsand incomplete questionnaire.	General linear model comparing literacy groups adjusting for age, education and monthly income	Cross-sectional nature makes it difficult to draw causal pathway between health literacy and rates of arthritis.Seven subjects were excluded because of their vision problems and incomplete questionnaire.
Rudd et al.(2009)[19]	134 consented and 127 of those were randomized.	Multivariate models were runwith and without adjustmentfor covariates that differedat baseline between the groups	There may have been a ceiling effect since participants had higher baseline education, literacy skills, satisfaction, adherence and attendance. This also limits generalizability. The average disease duration in this population was 17years thus they might have been very experienced with arthritis and arthritis care already.
Quinlan et al. (2010) [18]	27 of 157 (17%) did not participate dueto various reasons, it is possible that somerefused due to poor health literacy/inabilityto fill out the instruments	Stepwise regression with race, education, neighbourhood income, duration of disease,study location, age, healthknowledge, medications.	Use of self-modified version of TOFHLA, unclear if any validation
Swearingenet al. (2010)[20]	No attrition or refusals	Not reported	Socioeconomic status not described. Participants in this study were used to filling out questionnaires at each visit therefore their health literacy/skills completing questionnaires might be better than other population

BMI = body mass index.

Attrition bias resulted from patients being lost to follow-up, or unable to complete the measurement of health literacy. One study reported that 17% of participants were unable to complete the REALM. [12] Three studies reported attrition due to inability to read or illiteracy, [13], [14], [15] while another study reported losses due to incomplete questionnaires or visual impairment in participants. [17].

Three studies performed multivariate analysis to account for potential confounding variables in the association between low health literacy and health status or outcomes. [12], [16], [18] The relationships reported in other studies may potentially be affected by confounding factors.

Selective outcome reporting (favoring significant findings) is a possible source of bias as the cross-sectional surveys may have performed a wide range of measurements and analysis, but only a limited range reported within the available space for a published manuscript. Unlike prospective clinical trials, there is no facility for pre-registration of study protocols for observational studies, and we were unable to ascertain whether any outcomes had been added or omitted. There was considerable diversity in the measures of patient outcomes, ranging from global measures such as the MD-HAQ to specific rheumatologic scales (pain, function or Disease Activity Score DAS-28).

## Discussion

Our systematic review found no clear or consistent relationship between levels of health literacy and disease-specific outcome measures in older patients with chronic musculoskeletal conditions. However, a number of methodological weaknesses and the relatively small sample sizes may have hindered the assessment of any apparent association. A potential link was demonstrated in Kim’s study but there were major methodological limitations in this study when compared to the other four studies that reported no significant association between health literacy and musculoskeletal outcomes. [17] The data on healthcare utilization is also uncertain, although one study, with possible confounding factors, indicated that patients with low health literacy had more frequent hospital attendance. [14] In contrast, there is slightly more evidence of a relationship between low health literacy and poorer overall health status in patients with chronic musculoskeletal conditions.

A possible interpretation of our findings is that (functional) health literacy genuinely has no direct link with disease-specific measures or symptoms in older patients with chronic musculoskeletal conditions. Equally, the small sample sizes and lack of power may have hindered the detection of any true association. For instance, Type II error (failure to detect a genuine association) may have occurred because there were insufficient numbers of study participants with low health literacy. Absence of statistically significant findings in multivariate adjusted models may reflect both the lack of statistical power, and/or reduction of confounding influences. The inconclusive findings may also arise from differences or limitations in the current methods of measuring health literacy and clinical outcomes. For instance, patients with low health literacy may have difficulty in completing multiple items in disease-related questionnaires, and their responses may not have been accurately captured.

Validity of the individual studies should be considered when evaluating the evidence as a whole. Most of the included studies were cross-sectional in nature, and we cannot establish a causal relationship between the measures of health literacy and outcomes. Attrition or exclusion of participant with poor reading skills or visual impairment could lead to an unrepresentative dataset, with less representation from patients with low literacy.

The observational nature of the studies means that confounding is a threat to study validity and it may not have been possible to adequately adjust for confounders in the smaller studies. Patients with low health literacy had a higher rate of pain and functional limitation in Kim’s study but this could simply have stemmed from the significantly higher recorded prevalence of arthritis (type unspecified) in those with low health literacy. [17] The higher rate of arthritis here could potentially be related to other factors such as age, and previous occupation, given that the patients with low health literacy were older and were from a lower income group. In Gordon’s study, greater levels of anxiety and lower socio-economic status amongst patients with low health literacy may have had an important influence on the reported increase in the rate of hospital attendance. [14].

Heterogeneity of findings is likely due to the different populations evaluated, and inconsistencies amongst the measurement tools. Kim’s study used a modified Korean language specific version of TOFHLA. [17] However, lack of consistency in the tools for measuring health literacy may contribute to differences in estimated prevalence. For instance, while Buchbinder et al. reported that prevalence of low health literacy with TOFHLA (9%) or REALM (10%) was similar, [13] we noted that there were discrepant findings between the two methods of classifying health literacy. Here, five patients with inadequate/marginal health literacy on TOFHLA were judged to have adequate health literacy on REALM (scores of 65–66) whereas five patients classified as low health literacy on REALM (score <61) were judged ‘Adequate’ with TOFHLA. [13] Equally, the discrepant findings seen between the SILS tool and the REALM/S-TOFHLA in Hirsh’s study may be due to the poor performance and limited positive predictive value of SILS. [15] [21], [22] These methodological issues make it very difficult to provide a robust comparative interpretation of health literacy measures in different studies and populations.

One study looked specifically at individual perceptions of disease severity, and found that there were significant discrepancies in the views expressed by physicians as compared to their patients with low health literacy. [15] Differences in opinion could adversely affect patient-physician communication during clinical consultations, and may potentially account for Gordon et al’s finding that low health literacy was associated with greater anxiety levels and more frequent hospital visits. [14] There is some suggestion that an educational intervention may help improve mental health of patients with musculoskeletal disease, but this trial was limited by difficulty in recruiting and follow-up, with only short-term benefit apparent from the literacy intervention. [19].

We selected studies that focused on measures of functional health literacy, but there are likely to be a wide range of other individual factors that influence disease severity, and extent of symptoms and signs in patients. Patient symptoms and outcomes are an end measure which can be influenced by many factors. One could hypothesize that low health literacy skills may be associated with less knowledge about the disease or associated drug therapy, as well as greater anxiety and more medication errors, but this may not necessarily lead to deterioration in objective measures of disease control if low-literacy patients are attending the clinic more frequently as shown in Gordon’s study. [14] Studies need to focus on all these areas, and the role of important confounding factors.

The currently available health literacy measures have been criticized for being limited to functional health literacy. [23] Broader concepts of health literacy include cognitive and social skills, [24] while communicative/interactive health literacy reflects the ability to derive information and meaning from different forms of communication, and applying these in changing settings. Critical health literacy is thought to relate to the ability to appraise information and make decisions about one’s health and healthcare. [25] These aspects of health literacy need further research in order to determine their relationship (if any) to clinical outcomes.

In addition, current studies may not have adequately considered duration of illness as an important variable during participant selection. For instance, regular attendees at the rheumatology clinic may have become experienced at coping and managing specific symptoms, even though their functional health literacy scores are suggestive of low literacy. Moreover, significant differences between patients may not be easily detected if participants who provided written consent were mainly from the higher literacy level. We note also that presence of visual or cognitive impairment was an exclusion factor in some studies despite these very patients being potentially most at risk of health literacy problems and therefore important to include in any such study.

### Comparison with Other Studies

In 2004 Dewalt et al. conducted a systematic review of health literacy and health outcomes which was updated in 2011. [10]–[11] The authors concluded that low health literacy was associated with differential use of health care services, poorer ability to demonstrate taking medication properly and interpret medication labels and, amongst elderly persons, poorer overall health status and higher mortality. Our review looked at a different set of studies on health literacy, with the primary aim of evaluating disease-specific outcomes relating to older adults with chronic musculoskeletal conditions. In addition, we were particularly concerned about the potential risk of bias from confounding factors, and the methodological limitations in measurement of health literacy and disease outcomes. This may explain the somewhat less positive findings from our review as compared to the other systematic reviews. [10], [11].

### Limitations

Our review had some limitations. Our search was restricted to English-language articles, and we had to use a variety of synonyms for health literacy in the search as there were no consistent indexing terms amongst the electronic databases. Although this creates difficulty in retrieving studies on health literacy, [26] we believe that our broad search terms (with >1500 hits to be screened) should be sufficiently comprehensive. In order to reduce the risk of publication bias, we have included data from two separate PhD dissertations, [12], [18] although we are aware that the findings from one dissertation has subsequently been reported in journal manuscripts. [27], [28] We have had to rely on the published versions of most studies, where there is a potential for selective outcome reporting and publication bias. Our review focuses mainly on the chronic musculoskeletal conditions that have greatest population and health services burden (e.g. osteoarthritis and rheumatoid arthritis), and the findings may not be applicable to other less prevalent musculoskeletal conditions.

### Clinical and Policy Implications

The current evidence does not show any clear relationship between low health literacy and disease-specific outcomes or symptoms in older people with chronic musculoskeletal conditions. In the absence of a significant association, any proposed educational interventions to improve health literacy in patients with rheumatologic conditions would not necessarily show a beneficial impact (especially where only functional health literacy is being measured).

We believe that more robust research is needed, with appropriate statistical adjustment for confounding factors and wider, generalized recruitment of participants, preferably through adequately powered longitudinal cohort studies that can better discern cause and effect. There is also an important role for qualitative research in order to clarify the discrepancy that exists in judgment of health status between patients with low health literacy and their physicians. We also note the presence of some discrepant findings between different tools used in measuring health literacy, and are aware of discussion about specific rheumatology related instruments. New methods of measuring health literacy, beyond the functional level, are also required and there are a number of tools currently being evaluated. [29], [30], [31].

## Methods

### Search Strategy

We searched CINAHL, Embase, Medline, and PsycInfo for English-language articles from inception until January 2011. Full details of the search strings are given in Appendix S1. Reference lists of relevant articles (including other systematic reviews) were scanned for further articles. Citations were then independently screened for inclusion by two research associates (XW, IH). We also set up a monthly electronic alert from PubMed for new articles bearing the relevant search terms (last update February 2012).

### Study Selection and Eligibility Criteria

The initial screen looked at titles and abstracts, and excluded articles that clearly did not relate to health literacy and the chronic musculoskeletal conditions of interest listed below. Reviewers then read all potentially relevant articles in full to determine whether they met the selection criteria. Disagreements were resolved by discussion of all three reviewers.

Eligibility and relevance of articles was assessed based on detailed checking against the following criteria:

1. Includes older adults (mean or median age ≥50 years);
2. A validated measure of health literacy such as the Rapid Estimate of Adult Literacy in Medicine (REALM) or Test of Functional Health Literacy in Adults (TOFHLA), [7] or a health literacy scale specific to musculoskeletal conditions;
3. Chronic musculoskeletal disease – osteoporosis, rheumatoid arthritis, osteoarthritis were amongst the conditions evaluated in the study;
4. Assessed prevalence of health literacy, and/or analyzed relationship between health literacy and healthcare outcomes.

Both published and unpublished data (including abstracts, PhD dissertations) were eligible for inclusion.

### Data Extraction

Two investigators (IH, XW) independently extracted data on study design, sample size, participants’ characteristics, method of measuring health literacy and healthcare outcomes, and results from any quantitative analysis of the association between health literacy and outcomes. The extracted data was then checked by the third reviewer (YL), and agreement reached by consensus amongst the three reviewers on the finalized items.

### Validity Assessment/risk of Bias

The two investigators rated validity of the included studies and their assessments were discussed and agreed through consensus with the senior reviewer (YL) prior to being combined into a single table.

In order to evaluate the strength of the evidence, included studies were assessed with regard to five aspects of their design:

1. Selection of participants;
2. Measurement of health literacy including details on the tool used;
3. Assessment of healthcare outcomes, and whether the method used had been validated;
4. Methods used in assessing relationship or correlation between health literacy and outcomes, and how potential confounding factors were addressed;
5. Attrition bias, particularly loss to follow-up, missing data or incomplete reporting.

## Supporting Information

Appendix S1 **Search Terms.**
(DOC)
