# Supplementary material for: Impact of Health Literacy in Patients with Chronic Musculoskeletal Disease–Systematic Review
Source: PLoS One. 2012 Jul 6;7(7):e40210. doi: 10.1371/journal.pone.0040210 (PMC3391211; doi:10.1371/journal.pone.0040210)
Supplement: Appendix S1 — Search Terms. (DOC) [file pone.0040210.s001.doc]

# Appendix S1. Search terms

**Search Strategy for Ovid SP, with suffix .mp (mp=ti, ab, sh, hw, tn, ot, dm, mf, dv, kw, ps, rs, nm, ui). Limited to English language, Human.**

**Literacy terms:** health-literacy OR numeracy OR health-behaviour OR health-education OR Health-attitude OR Health-knowledge OR Rapid-Assessment-of-Adult-Literacy-in-Medicine OR TOFHLA OR Test-of-Functional-Health-Literacy-Assessment OR Health-Activity-Literacy-Scale OR REALM

**AND**

**Disease terms:** arthritis OR rheumatoid OR osteoporosis OR osteoarthritis

**Monthly Automated electronic updates from PubMed (up to February 2012) for any new articles based on the following search terms**

("health literacy"[MeSH Terms] OR ("health"[All Fields] AND "literacy"[All Fields]) OR "health literacy"[All Fields]) AND (("osteoporosis, postmenopausal"[MeSH Terms] OR ("osteoporosis"[All Fields] AND "postmenopausal"[All Fields]) OR "postmenopausal osteoporosis"[All Fields] OR "osteoporosis"[All Fields] OR "osteoporosis"[MeSH Terms]) OR ("arthritis"[MeSH Terms] OR "arthritis"[All Fields]) OR osteoarthrities[All Fields] OR musculoskeletal[All Fields])
